# Covid-19 Pandemic: Maximizing Future Vaccination Treatments Considering Aging and Frailty

**DOI:** 10.3389/fmed.2020.558835

**Published:** 2020-09-18

**Authors:** Sara Palermo

**Affiliations:** ^1^Department of Psychology, University of Turin, Turin, Italy; ^2^European Innovation Partnership on Active and Healthy Aging, Bruxelles, Belgium

**Keywords:** COVID-19, ageotype, frailty, vaccination, adherence to treatment, immunosenecence, inflammaging, immunology

## Abstract

The COVID-19 pandemic is proving to be a multiplier of inequalities. Especially toward the elderly population. A voiceless scream that comes from geriatrics, nursing homes, hospices from all over Italy. They call it the silent massacre: from North to South, the bulletin of coronavirus positive—or already deceased—elderly people continues to grow exponentially without a chance to counter it. Population aging and chronicity are a question that needs to be addressed. Frailty is the most challenging expression of population aging, with major consequences for public health and clinical practice. It is a geriatric syndrome which consists in a state of higher vulnerability to stressors attributed to a lower homeostatic reserve due to an age-related multisystem physiological change. People over 60, and especially over 80, are particularly vulnerable to severe or fatal infection. Moreover, the age-related dysregulation of the immune system in the elderly (i.e., immunosenescence and inflammaging) results in poorer responses to vaccination. Physical frailty is an effective health indicator and it has previously shown to predict the response to the seasonal flu vaccine. These findings suggest that assessing frailty in the elderly may identify those who are less likely to respond to immunization and be at higher risk for COVID-19 and its complications. Moreover, cognitive frailty and neurocognitive disorders, mental health and reduced awareness of illness negatively impact on adherence to complex medication regimens among elderly patients. A worldwide research and development blueprint have been initiated to accelerate the development of vaccines and therapeutics for the COVID-19 outbreak. Considered the above, I suggest the importance to consider aging in thinking about future Civud-19 vaccination and treatment, focusing on the possible impact of physical and cognitive frailty.

## Introduction

COVID-19 had such a high transmission rate in Italian nursing homes—even in the regions least affected by the virus—that they were considered “contagion multipliers” from the beginning of the pandemic. To shed light on the matter is the “National Survey on COVID-19 contagion in residential and social-health facilities” by the “Istituto Superiore di Sanità” (ISS, https://www.iss.it/en/home), which is the main center for research, control and technical-scientific advice on public health in Italy. ISS reports 3,859 deaths since 1 February, with 133 patients tested positive for Covid-19 and 1,310 elderly with flue-similar symptoms (1). Despite the drama of these data, national epidemiological data need to be considered to understand how devastating the impact of infection on the elderly is.

The ISS report -based on available data on April 13th, 2020 (18,641 deaths)—warned that the mean age of patients dying for SARS-CoV-2 infection was 79 years (median 80, range 5–100, IQR 73–86). Women dying for SARS-CoV-2 infection had an older age than men (median age women 83–median age men 79). An increase in lethality is observed with increasing age. Lethality is higher in male subjects in all age groups, except for the age group over 90 years old. Moreover, pre-existing conditions were usually observed in SARS-CoV-2 positive deceased patients. This information is not complete. There is the gray number of patients never screened to which is added the number of elderlies for whom the potential infection has never been reported. Probably, many domestic deaths could be the consequence of the virus. This is not just an Italian problem.

COVID-19 is causing “hidden deaths” —elders left to die in their beds or hidden from official death statistics—across Europe. Over 95% of people who died of SARS-CoV-2 in Europe were over 60, according to WHO. More than four in five of those people had at least one other chronic underlying condition. Half of coronavirus deaths happen in care homes. Snapshot data on April 12th from 5 European countries suggest that care home residents have so far accounted for between 42 and 57% of all deaths related to COVID-19, according to the London School of Economics.

This general framework imposes a reasoning about the impact of the various types of aging not only on the possibility of incurring the infection but also on the prognosis and efficacy of potential treatments. At the beginning of May, Europe decided to do its part by accepting the WHO's invitation to join the “Access to COVID-19 Tools (ACT) Accelerator” initiative and investing in the development of diagnostic tests, drugs and vaccines against coronavirus. The proposed perspective article can stimulate further interest about frailty implications for the development of new vaccine formulations and vaccination protocols in case of pandemic emergencies within the reader audience. I presented here a different take on an existing issue that has important clinical implications but has been neglected in the early stages of the pandemic.

## Global Population Aging and Its Impact on Public Health

The world's population is aging. In 2018, people aged 65 and over exceeded children under the age of five. In 2019, one in 11 people was over 65 (9%). This ratio could move to 1 in 6 people (16%) by 2050. An even more important fact: the number of people aged 80 and over is expected to triple from 143 million in 2019 to 426 million in 2050 (2).

To date, the European Union consists of the over 500 million people, of whom about 100 million are elderly people (3). While increasing life expectancy is considered a major achievement, it has a significant impact on public health since the expense to be incurred depend on the interaction between individual determinants (age, mortality, disability, and health) and social determinants (national income, technology, and wages) (4).

Progressively there has been an epidemiological transition from infectious and disabling diseases to chronic-degenerative diseases (5). Indeed, population aging strongly increases expenditures on long-term care (6). Elderly people are also large medicines consumers, while a lot of older adults end up suffering from problems related to medication. The European Medicine Agency (EMA) ensures that the medicines used by older people are of high quality and are studied appropriately in the older population, throughout the medicinal product lifecycle. For that reason, the Clinical Trials Regulation (EC) No. 536/2014 states that “*in order to improve treatments available for vulnerable groups such as frail or older people, people suffering from multiple chronic conditions, and people affected by mental health disorders, medicinal products which are likely to be of significant clinical value should be fully and appropriately studied for their effects in these specific groups, including as regards requirements related to their specific characteristics and the protection of the health and well-being of subjects belonging to these groups*.” Indeed, EMA develops scientific guidelines to help medicine developers address the specific requirements of older people in their medicine development programs, including in the design and conduct of clinical trials. EMA disclosed a reflection paper on “Physical frailty: instruments for baseline characterization of older populations in clinical trials” (7), actively recognizing the importance of considering the various types of aging when experimenting and developing new pharmacological treatments. The value of vaccines for the elderly should be based on *efficacy*, the capacity to confer protection against a specific infection; *effectiveness*, the capacity to generally improve health by avoiding other related diseases. To enhance both parameters, it is necessary to better understand aging.

## Aging, “Ageotypes” and Frailty

There is no single way of aging, but there are as many different aging processes as there are humans. However, some main directions can be identified. Aging is a gradual and continuous process of natural mutation that begins in early adulthood. During the first year of middle age, many bodily functions begin a gradual decline. The life-span perspective recognizes changes in the functional state as characteristic of the human being aging process (8). Such changes are considered normal and are sometimes called *pure aging*. (9). *Successful aging* refers to the postponement or reduction of the unwanted effects related to advancing age. The main features of successful aging are the maintenance of physical health, an active and autonomous life; a full and satisfying emotional-relational life; prevention of ailments and disabilities. This perspective also applies to the neuropsychological domain: *successful cognitive aging* refers to people whose physical health may or may not be good, but whose cognitive profile remains exceptional; *typical cognitive aging* refers to people who experience a slow loss of cognitive efficiency that does not result in a neurocognitive disorder and whose distinctive feature is the reduction of mental processing speed (8).

### Ageotypes

It is a common opinion that aging is the main risk factor for many chronic diseases. Recently, different types of aging patterns (the so-called “ageotypes”) have been identified, based on the molecular pathways that changed over time (10). Four distinct biological pathways seem to be possible: metabolic (relating to the build-up and breakdown of substances in the body), immune (relating to immune responses), hepatic (relating to liver function), and nephrotic (relating to kidney function) (10). Therefore, ageotype may provide a molecular assessment of individual aging, reflective of personal lifestyle and medical history; it can help people focus on health risk factors and find areas where they are most likely to encounter problems down the line; it may ultimately be useful in monitoring and intervening in the aging process (10). Multimorbidity and polypharmacotherapy weakens the body and can predispose to accelerated aging, with an increase in disability, hospitalization, institutionalization, and mortality rate.

### Frailty

Frailty is certainly the most problematic expression of the aging population (11). It can be defined as a dynamic condition of increased vulnerability, which reflects age-related multi-systemic pathophysiological changes, associated with an increased risk of negative outcomes, such as institutionalization, hospitalization, and death (12). Frailty in the elderly is an integrated (13) and multidimensional (14) condition in which biological, functional, psychological, and social assets interact with each other, determining and characterizing fragility (15). To date, there is no unequivocal and recognized operational definition of frailty. EIP-AHA experts have identified two main approaches: the first concerns physical determinants (biomedical approach), while the second considers biological, cognitive, psychological, and socio-economic factors (bio-psycho-social approach). Indeed, a reliable assessment cannot be separated from the analysis of the affective, cognitive, and relational components (11). In support of this interpretation, the Sunfrail project (16) has defined frailty as a dynamic state that concerns the loss in one or more domains:

- Physical: weight loss, slowness, reduced physical activity, strength, and endurance.
- Psychological: cognition, mood, coping strategies.
- Socio-economic: social relationships, social support, income capacity.

Some scientific societies are recommending assessing frailty in patients with COVID-19 infection to guide their triage and the results seem to be promising (17). Considering scientific literature, frailty was only investigated regarding its association with overall mortality, hospital contagion, intensive care unit admission rates, and disease phenotypes (18). According to a recent systematic scoping review, specific interventions in relation to frailty or its impact on COVID-19 treatments have not been evaluated yet (18).

## Frailty and Immunosenescence

Aging leads to a progressive declining competence of the immune system, resulting in increased susceptibility to infectious diseases. Flu is among the main causes of infectious death—and the most important agent of respiratory disease outbreaks—among the elderly (19). Indeed, morbidity and mortality due to respiratory infections increase in subjects ≥65-years old (20). Often subtle clinical manifestations may not be recognized initially in the elderly, preventing timely administration of antiviral treatment (19–21). To compound the situation, the effectiveness of vaccination is often reduced due to *immunosenescence* (21). Aging results in a weakening in immune competence, which is characterized by increased autoimmunity, inability to maintain immunity to latent infections, increased risk of chronic inflammatory diseases and infections; disfavored vaccine efficacy (21).

Frailty afflicts the immune system, more than would be expected because of aging (22). A dysregulated immune system—characterized by amplified immunological markers of T-cell senescence and intensified inflammation—has been identified in frailty elderly (22). Immunosenescence and frailty are commonly described in older adults and appear to share common inflammatory factors (23). It is unclear whether they are separate entities that occur due to coincident or potentially confounding factors, or if they are connected by the same underlying cellular mechanisms (23). However, it is possible that they support each other, triggering a deteriorating process that profoundly affects health and the ability to adapt to viral infections in the elderly.

## Frailty and Inflammaging

Aging has been associated with an increase of inflammatory biomarkers (24). This age-related fluctuation of the inflammatory mediators is referred to as “inflammaging.” This concept refers to the connection between the aging process and a type of chronic low-intensity inflammation that has no visible (latent) symptoms but that produces systemic effects on the whole organism (25, 26). Inflammaging is considered a major immunological characteristic of the elderly and an etiological agent for age-related pathologies (25–28).

Elevated levels of inflammatory cytokines in older adults are suggestive of inflammaging (29). Inflammation plays a central role in physical frailty. Not only changes in hormone and inflammatory cytokine levels may mediate frailty among postmenopausal women (30), but a mouse model of frailty and chronic inflammatory pathway activation demonstrated the upregulation of numerous proinflammatory cytokines (31). The hypothesis that inflammation affects multimorbidity and frailty by increasing catabolism, inhibiting growth factors, and interfering with homeostatic signaling is being considered by researchers (32). Although pending further confirmation, inflammatory biomarkers could support diagnosis, prognosis, and therapeutic decisions in frail elderly (24).

It is important to note that inflammaging causes profound changes in crucial components of the innate immune system, which are related to an increased risk of infection and increased infection-related mortality (33). In addition, the importance of molecules that contain damage-associated molecular patterns is emerging to activate innate immune cells and maintain the state of inflammaging (34). Between immunosenescence and inflammaging there is a mutually maintained state where immunosenescence is induced by inflammaging and vice-versa (34). Increased production of inflammatory mediators contributes to the decrease of the adaptive immune response and, eventually, to immunosenescence (34). In contrast, the decrease of the adaptive immune response reinforces the stimulation of the innate immune response leading to inflammaging (34).

## Frailty and Multimorbidity

Frailty and multimorbidity are not synonyms (35). Quoting Villacampa-Fernández and colleagues [(35), p. 31]: “*frailty identifies the increased vulnerability to stressors due to a dynamic, non-linear, and multidimensional depletion of physiological reserve and redundancy, whereas multimorbidity refers to the coexistence of two or more clinically manifest chronic diseases*.”

Elevated blood levels of pro-inflammatory markers are a powerful risk factor for multimorbidity and predict its future rates of change (32). Moreover, multiple chronic conditions are a significant contributor to pre-existing frailty (36). A recent meta-analysis found that the prevalence of multimorbidity in frailty elderly was 72% and the prevalence of frailty among multimorbid individuals was 16% (37). Moreover, the number of chronic diseases mediates the relationship between sex and the number of frailty components, and the latter mediate the relationship between the number of diseases and disability (38).

Considering vaccination, increasing frailty or the coexistence of multiple chronic conditions contributes to the loss of vaccine effectiveness (39).

## Frailty and Mental Health

Much of the research literature on frailty has focused on physical health. However, mental health including cognition, personal well-being, and social interactions are as important as those related to physical illness and disability (40). Frailty may be relevant in identifying older people at risk of deteriorating mental health (41). Indeed, for each additional deficit-defining frailty, odds of psychiatric illness increased (42). Moreover, while an association with increased emotional distress has been found at the earliest stage of pre-frailty, once frailty develops there is a higher likelihood of clinically significant depression and anxiety (41).

Frail elderly individuals were significantly more likely to have cognitive decline, memory decline, and sarcopenia (43). Cognitive frailty specifically refers to the co-occurrence of mild cognitive impairment and physical frailty in the absence of a major neurocognitive disorder diagnosis (44). Interestingly, individual indicators of frailty have been independently associated with cognitive function (45). Processing speed, executive function, and attention have been associated with weak grip strength and slow walking speed (45). Moreover, researchers found that frailty was directly associated with poorer executive function and worse sleep quality, which was also associated with worse processing speed (40). The presence of physical and/or cognitive frailty in the elderly increases the risk of negative outcomes and leads to greater use of health and care services (11).

The interaction between mental health and the immune system is the subject of study in psychoneuroimmunology, which highlights the overcoming of brain-mind-body trichonomy (46). Not only the immune and emotional systems mirror each other, but both the immunological and emotional responses are dynamic and continuously changing (47). Clinical studies point to roles for the immune system in psychiatric diseases, while basic science has revealed that the brain has an active and multi-cellular resident immune system that interacts with peripheral immunity and impacts behavior (48). Conversely, mental health disorders can affect the immune system. Indeed, relatively intense, or chronic stressors can produce *immunosuppression* (49).

Pathogens of different nature, changes in environmental conditions, and significant life events cause an exacerbated or dysfunctional compensatory immune or emotional response in patients suffering from emotional or immunological disorders (47). For example, major depressive disorder (MDD) has a marked effect on the immune system (50). Indeed, serum levels of interleukin-5 are elevated in persons with MDD. Interleukin-5 has been previously associated with immune system T helper cells and conditions such as allergy. A higher risk of depression and of prevalence of underdiagnosed major neurocognitive disorders have been found in older frail outpatients (51).

## Frailty, Vaccine Efficacy and Effectiveness

Vaccines are one of the most effective therapeutic interventions against infectious diseases (52). Nonetheless, the most widely used vaccines would appear to be less immunogenic in the elderly than in young adults (53). A useful example in the current pandemic context: the ability of the flu vaccine to induce protection is age-related, with an efficacy between 70 and 90% in children and adults (53). These percentages drop to 30–50% at best for subjects ≥65-years old (53). The estimated vaccine efficacy was of 23% in volunteers aged 70 years or older (54).

Elderly are less responsive to vaccination prevention, probably due to the combined effect of immunosenescence and inflammaging. Both are associated with frailty, however the impact of frailty on vaccine efficacy and effectiveness is uncertain. Hypothetically lower efficacy among vaccinated frail elderly has been suggested, as proven by increasing all-cause mortality rates with increasingly impaired functional status (55). Frailty has been associated with impairment in TIV-induced strain-specific immunity to influenza, increased rates of influenza-like illness and laboratory-confirmed influenza infections (56). A more recent study found that pre-vaccination strain-specific immunity is strongly associated with post-vaccination responses in frailty, with the highest associations in microneutralization titers in robust elderly (57). Vaccine efficacy seems to be remarkably distinctive across levels of frailty (58). It has been found to be optimal among robust elderly, lower among prefrail and frail subjects, and not recognizable in the frailest ones (58). These findings suggest the importance of accounting for frailty when assessing the impact of influenza vaccines (58).

## An Immunobiography-Based Approach To Vaccine Formulation

**Figure 1:**
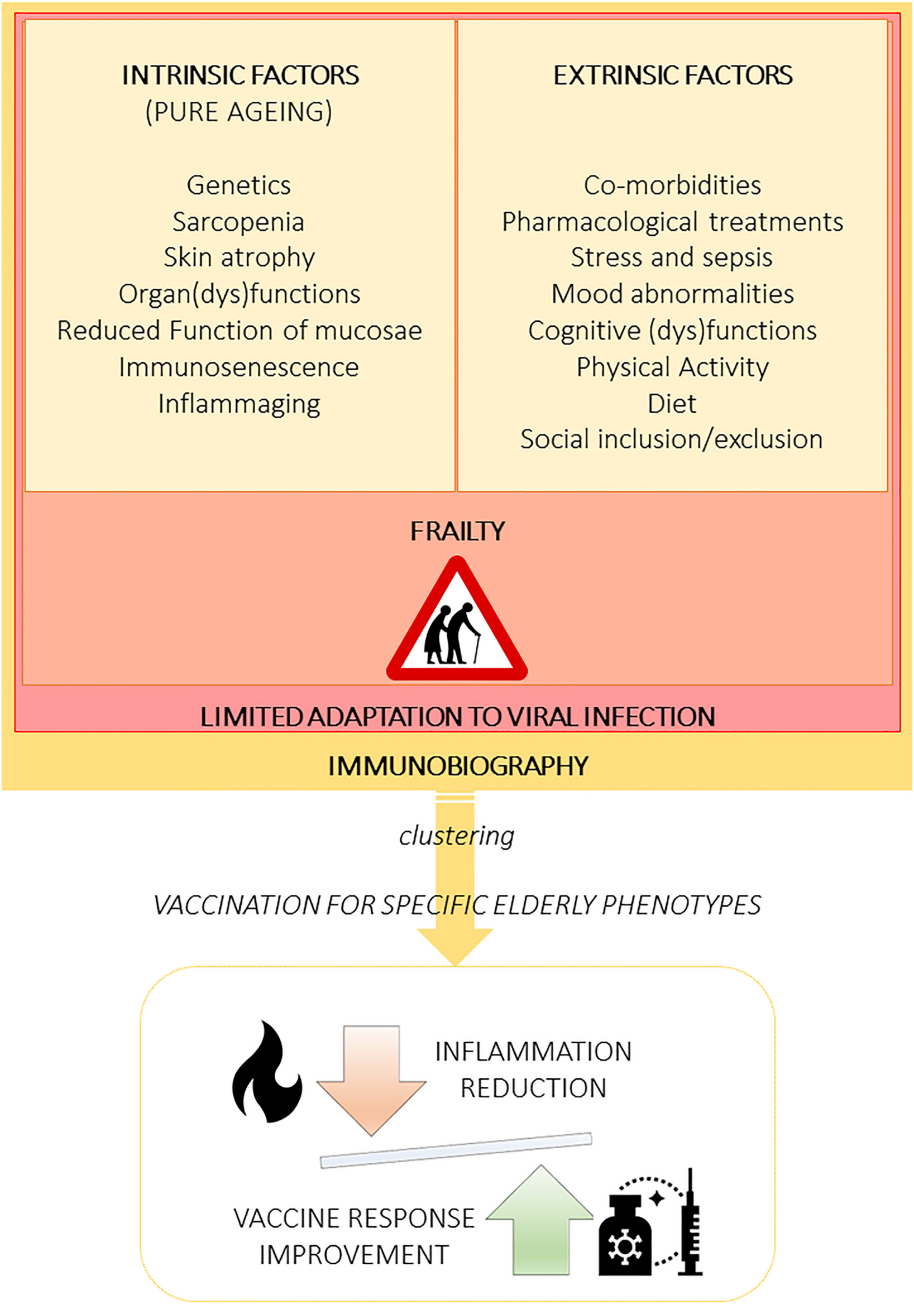
Aging is influenced by multifaceted extrinsic and intrinsic factors leading to several elderly phenotypes that must be recognized and clustered to specifically design vaccine formulations, including adjuvants. The immunobiography approach could inform the stratification of elderly subjects and guide the implementation of vaccination strategies designed for specific elderly population clusters. Importantly, vaccines should be created to optimally balance immune stimulation and inflammation.

The search for formulations of new vaccines should consider the intrinsic (secondary to pure aging) and extrinsic factors that influence deterioration in immune response (59). Understanding their contributions can reveal the mechanisms that lead to the progressive decline of immune competence with age and to identify the “immunobiography” of each subject (52). Follow this approach could allow developing novel and improved vaccines for the older adults. Specifically, it can help identify different aging clusters would allow to develop vaccines according to the geriatric phenotype, facilitating inflammation reduction and improving vaccine response (Figure 1).

## Aging Clusters, Vaccine Formulations and Vaccination Protocols

It is of primary importance to design vaccine formulations and vaccination protocols specifically tailored on the elderly (60). It is therefore necessary to consider possible aging clusters, immunosenescence, and inflammaging. This priority seems to be appropriate for the European vaccine development roadmap promoted by the Innovation Partnership for a vaccine roadmap in Europe (IPROVE) (60).

**Figure 2:**
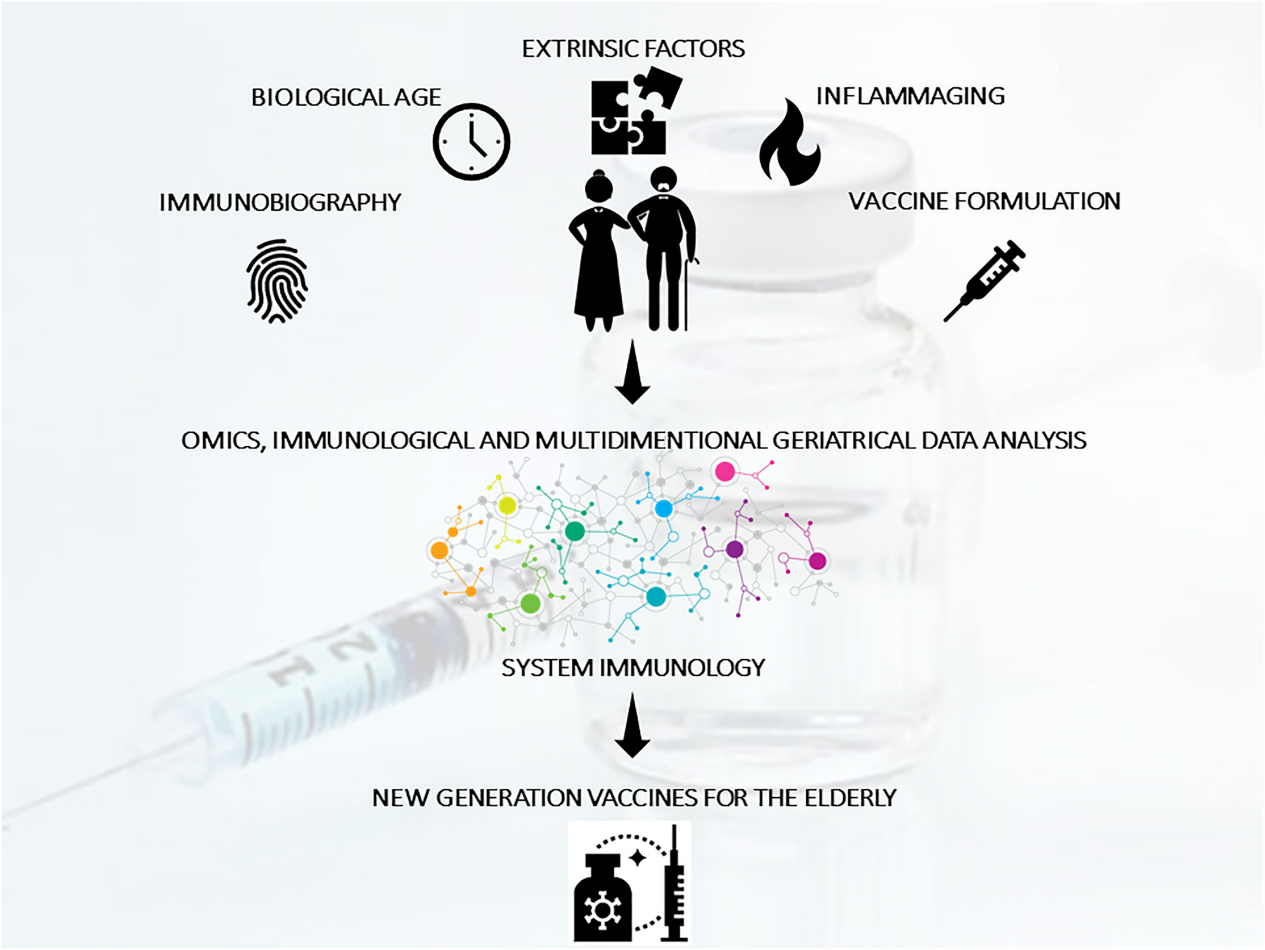
The improvement of vaccination strategies for the elderly should consider mechanism of action specifically suited to act in the context of immunosenescence/inflammaging and taking in consideration different clusters of elderly and individual immunobiography. With this aim, geriatric, immunological, clinical, and omics data (generated from clinical studies of vaccination in the elderly) should be integrated using a systems vaccinology approach to guide the design of next generation vaccines specifically tailored for the elderly population.

Promising research routes to improve the value of vaccines should consist of: (a) the inclusion of a comprehensive geriatric approach to aging; (b) the valorization of peculiar aging phenotypes and immunobiographies; (c) the integration of geriatric, immunological, clinical, and omics data according to a systems vaccinology approach to guide the design piof next generation vaccines and adjuvants specifically tailored for the elderly (27) (Figure 2).

## COVID-19, SARS-CoV-1 and MERS-CoV: Lessons From The Previous Two Pandemics

The pathogenic Middle East respiratory syndrome coronavirus (MERS-CoV), severe acute respiratory syndrome coronavirus (SARS-CoV-1), and COVID-19 coronavirus (SARS-CoV-2) have all emerged into the human population over the last two decades and resulted in substantial numbers of deaths. The genome of COVID-19 partially resembled SARS-CoV-1 and MERS-CoV and indicated a bat origin (61). All three are enveloped, positive-sense, single-stranded RNA viruses that belong to the family Coronavirdiae, and are known to cause acute respiratory, hepatic, and neurological diseases with varying severity. The most common presenting symptom is fever, followed by cough, sore throat and dyspnoea (62).

Although related to SARS-CoV-1 and MERS-CoV, COVID-19 shows some peculiar pathogenetic, epidemiological and clinical features which to date are not completely understood (62). COVID-19 generally has a less severe clinical picture, and thus it can spread in the community more easily than MERS and SARS, which have frequently been reported in the nosocomial setting. Consistent with its clinical manifestations, COVID-19 has a fatality rate of 2.3%, lower than that of SARS (9.5%) and much lower than that of MERS (34.4%).

To date, no specific antiviral treatment or vaccine is available for treatment of COVID-19. Previous therapies targeting SARS-CoV and MERS-CoV may accelerate the development of COVID-19 treatment because of their structural and genomic similarities (61). Those coronaviruses make use of a large envelope protein called spike (S) to engage host cell receptors and catalyze membrane fusion. Because of the vital role that these S proteins play, they represent a vulnerable target for the development of treatments (63). Due to the indispensable role of the S-protein, therapies and vaccine exploration targeting S-protein-ACE2 interaction may be very promising (61). Previous experience from SARS-CoV-1 and MERS-CoV treatments development have targeted angiotensin receptor blockers, gene silencing technologies, glycoprotein epitopes, and monoclonal antibodies, which may be useful for COVID-19 too (64).

In the context of coronavirus, vaccine development started seriously after the SARS-CoV and MERS-CoV outbreaks, providing alternative approaches of applying subunit vaccines, whole inactivated virus, vectored, and live attenuated virus vaccines (64). Indeed, if any cross-reactive epitopes were identified between COVID-19 and SARS-CoV, previous vaccine for SARS-CoV might be re-utilized to facilitate COVID-19 vaccine development (65).

## COVID-19: Mutation Rate and Its Implication For Vaccine Formulation

All viruses evolve over time, accumulating mutations that replicate imperfectly within the cells of a host in huge numbers and then spread into a population, with some of these mutations persisting through natural selection. SARS-CoV-2 is a single-stranded RNA virus. Such viruses are notorious for high mutation rates. One variable shaping COVID-19 immunotherapy efficacy is how quickly the coronavirus mutates. A faster rate of mutation would increase the likelihood that the vaccine would not generate an effective immune response.

Only as an example, 149 sites of mutations were identified across the genome of 103 sequenced strains of SARS-CoV-2 in in Wuhan (China), and the virus had evolved into two subtypes, termed L and S subtype (66). The two subtypes showed great differences in geographical distribution, transmission ability, and severity of disease. The L type is more prevalent than the S type (70 vs. 30%) and it might be more aggressive and spread more quickly. Type S, which is evolutionally older and less aggressive, has increased in relative frequency over time probably due to weaker selective pressure (66).

This and similar scenarios add up more obstacles for vaccine design. Nevertheless, evidence seems to show that SARS-CoV-2 may have a relatively slow mutation rate for an RNA virus, increasing the chances that a vaccine would offer long-term protection (67). Currently, there are six strains of COVID-19. The original one is the L strain, that appeared in Wuhan in December 2019. Its first mutation—the S strain—appeared at the beginning of 2020, while, since mid-January 2020, we have had strains V and G. To date strain G is the most widespread: it mutated into strains GR and GH at the end of February 2020 (67). The small number of genetic differences between the original strain of the COVID-19 from Wuhan and those currently circulating indicates that a vaccine may likely offer lasting immunity (67).

## An Additional Factor: Adherence To Vaccination Treatment

To be successful in reducing the prevalence and incidence of a disease, vaccination programs rely on a high uptake level. Some of the elderly population remain reluctant to take advantage of the offer of vaccination (68). Compliance with vaccine recommendations lowers getting older and the burden of vaccine-preventable diseases remains high in the elderly and the oldest old (26).

Adherence rates have been shown to be related to age, race, sex, illness perceptions, and geographic residence (69). Moreover, depression, social exclusion, and low quality of life have a negative impact (70). Also, polypharmacy is associated with poorer adherence to treatment (71). Several other factors have been identified as contributors to low vaccine coverage in the elderly, including logistic factors such as ease of access and convenience, cultural attitudes, health literacy, and vaccine hesitancy (72). Hesitancy was defined by the WHO as the delay or refusal of vaccination despite the availability of vaccine services. Vaccination uptake has been associated also with perceived vaccination effectiveness, and the perceived likelihood or severity of vaccination side effects (73). For older adults, additional factors influencing vaccination uptake were underlying chronic diseases, and recent advice through physician consultation (73).

### Frailty and Adherence to Treatment

Higher level of frailty in the elderly has been proposed to be a determinant of lower adherence (74). A decrease in physical activity and difficulty in walking negatively affect adherence (71, 75). Additionally, some psychosocial frailty determinants have a strong influence: mourning and loneliness, a serious illness of some evidence argues that cognitive frailty (traditionally referred to as cognitive impairment) may also negatively affect adherence to treatment protocols and medication regimes in the elderly (76–79). Indeed, poor adherence in cognitive frailty patients seems to range from 10.7 to 38% (80). Mild cognitive impairment (that it means cognitive frailty) has been recently associated with lower adherence (45, 81). Cognitive deficits related with physical frailty (attention/mental flexibility, working memory, and executive functions) (82–84) seem to be able to predict poor adherence to treatment (41). A further factor—associated with the previous ones—which significantly impacts on adherence to treatment is a reduction in self-awareness (85, 86). Reduced self-awareness has been found to be associated with a decline in help-seeking behavior and compliance with medical treatment, presumably because of a reduction in motivation (85). In particular, the uptake of influenza vaccines has been found to be largely driven by the risk perception of the disease (87).

### How to Improve Vaccine Uptake

Getting vaccinated is therefore the result of a complex series of behaviors, all of which are contingent on an interlocking system of thoughts and beliefs, people, funding, policies, and permissions (88). This complex interlocking system is vulnerable to mental illness and neuropsychological disorders. Particularly impacting are executive-metacognitive dysfunctions and alterations in mood (anxiety, apathy, and depression). A comprehensive appreciation of the physical, neuropsychiatric, and neuropsychological profile of the patient influence on non-adherence is necessary for clinicians to improve the quality of care. According to Ecarnot et al. [(72), p. 234], “*strategies to improve vaccine uptake can target all the components underpinning low coverage, and include technology and communication-based strategies, physician-centered approaches, targeting healthcare workers for influenza vaccination, system-based factors, improved vaccine efficacy, and above all, political will and leadership*.”

## Conclusions

Each elder reflects the uniqueness of his/her own life, therefore requiring personalized assessment and management tools, pharmacological treatments, and care. To face the challenge of an aging population, public health must consider the existence of several aging patterns and promptly adapt to the current needs, especially in the event of emergency situations such as COVID-19 pandemic. A vaccine would offer lasting protection. The entire scientific community is working on a vaccine against Covid-19. Over 100 projects are under development. An incredible result if you think that only 3 months have passed since the exact sequence of Sars-Cov-2 was known. Nevertheless, the rate of coronavirus mutation hinders the vaccination program. Even vaccine might work against infectious disease, the efficacy was not certain because of its mutation rate of RNA virus. These mutations, however, do not impinge on the process of developing effective vaccines or weaken protection from a vaccine (67).

Starting next autumn, the possible return of SARS-CoV-2 will add to the seasonal flu. Vaccinating elderly people will be essential to have fewer people hospitalized for flu complications, to easily distinguish the latter from COVID-19 and to prevent effectively COVID-19 infections. However, vaccines being designed today are not going to be effective enough for the people who need them most: older adults. This perspective article aims to stimulate research to demonstrate whether the assessment of frailty in the elderly and targeted vaccination treatment will contribute to improve individual health and the sustainability of health-care systems. Indeed, vaccinations must be considered a fundamental medical, social, and economic intervention. With this aim, some promising routes that research can take to improve the efficacy and effectiveness of vaccinations in the elderly are shortly presented. The focus is given to the association among ageotypes, frailty, comorbidity, mental illness, immune system, and implications for vaccination. Although crucial advancement has been made in recognizing the mechanisms underlying age-related immune response decline to infections (and vaccinations), some knowledge gaps remain in basic and translational research and clinical practice. The progressive increase of the elderly population imposes new strategies to guarantee sustained health and well-being. The development of better and/or new vaccines against pathogens affecting the elderly is a fundamental intervention to achieve this goal. The covid-19 epidemic presents itself as an opportunity to rethink the strategies applied so far. Ongoing initiatives are expected to improve knowledge of COVID-19 interaction with frailty and to promote patient-centered approaches, also in the field of vaccine testing.

## Data Availability Statement

The original contributions presented in the study are included in the article/supplementary material, further inquiries can be directed to the corresponding author.

## Author Contributions

SP conceived the content of this perspective article, wrote the manuscript, and produced infographics.

## Conflict of Interest

The author declares that the research was conducted in the absence of any commercial or financial relationships that could be construed as a potential conflict of interest.
